# Supplementary material for: Relationships between Participation in Volunteer-Managed Exercises, Distance to Exercise Facilities, and Interpersonal Social Networks in Older Adults: A Cross-Sectional Study in Japan
Source: Int J Environ Res Public Health. 2021 Nov 13;18(22):11944. doi: 10.3390/ijerph182211944 (PMC8623852; doi:10.3390/ijerph182211944)
Supplement: Supplementary file 1 [file ijerph-18-11944-s001.zip › ijerph-1448108-supplementary.pdf]

Table S1. Characteristics of participants in volunteer-managed exercises.

		Silver-rehabili taisou exercise	Square-stepping exercise	Both exercises
		n = 734	n = 201	n = 364
Sex (female)		67.0	80.1	82.4*
Age (years)	Continuous	74<br>(69-78)	70<br>(67-74)	72*<br>(69-76)
	65–74	54.1	75.6	65.4*
	75+	45.9	24.4	34.6
Senior high school or more		71.3	79.6	78.8*
Living alone		86.2	86.6	83.8
Subjective economic status	Poor	13.2	11.4	9.3
	Normal	75.1	80.1	77.5
	Good	11.7	8.5	13.2
Arthralgia or neuralgia (yes)		26.3	21.4	27.5
Population density (n/km^2)		1,222.9<br>(438.3-1,673.8)	1,369.2<br>(594.2-1,778.5)	1,228.1<br>(514.3-1,664.4)
Main mode of travel	Car (driving)	59.7	68.7	61.6*
	Car (passenger)	17.7	13.4	11.5
	Cycling or walking	22.6	17.9	26.9
Weak interpersonal social network		4.5	5.0	3.0
Distance to exercise facility (m)		782.5<br>(414.6-1,216.8)	935.2<br>(534.0-1,826.6)	651.1*<br>(333.8-1,013.1)

Note. Values are median (25th, 75th percentile) or percentage. The p-value was obtained using the Kruskal-Wallis test and chi-square test between participating exercises. *$p < .05$.
